# Sociodemographic data collection for health equity measurement: a mixed methods study examining public opinions

**DOI:** 10.1186/1475-9276-12-75

**Published:** 2013-08-30

**Authors:** Maritt Kirst, Ketan Shankardass, Sivan Bomze, Aisha Lofters, Carlos Quiñonez

**Affiliations:** 1 Centre for Research on Inner City Health, Keenan Research Centre in the Li Ka Shing Knowledge Institute, St. Michael’s Hospital, Toronto, Canada; 2 Department of Psychology, Wilfrid Laurier University, Waterloo, Canada; 3 Department of Sociology, Syracuse University, Syracuse, USA; 4 Department of Family Medicine, University of Toronto, Toronto, Canada; 5 Faculty of Dentistry, University of Toronto, Toronto, Canada

**Keywords:** Health inequalities, Data collection, Public opinion

## Abstract

Monitoring inequalities in healthcare is increasingly being recognized as a key first step in providing equitable access to quality care. However, the detailed sociodemographic data that are necessary for monitoring are currently not routinely collected from patients in many jurisdictions. We undertook a mixed methods study to generate a more in-depth understanding of public opinion on the collection of patient sociodemographic information in healthcare settings for equity monitoring purposes in Ontario, Canada. The study included a provincial survey of 1,306 Ontarians, and in-depth interviews with a sample of 34 individuals. Forty percent of survey participants disagreed that it was important for information to be collected in healthcare settings for equity monitoring. While there was a high level of support for the collection of language, a relatively large proportion of survey participants felt uncomfortable disclosing household income (67%), sexual orientation (40%) and educational background (38%). Variation in perceived importance and comfort with the collection of various types of information was observed among different survey participant subgroups. Many in-depth interview participants were also unsure of the importance of the collection of sociodemographic information in healthcare settings and expressed concerns related to potential discrimination and misuse of this information. Study findings highlight that there is considerable concern regarding disclosure of such information in healthcare settings among Ontarians and a lack of awareness of its purpose that may impede future collection of such information. These issues point to the need for increased education for the public on the purpose of sociodemographic data collection as a strategy to address this problem, and the use of data collection strategies that reduce discomfort with disclosure in healthcare settings.

## Introduction

Monitoring inequalities in healthcare is increasingly being recognized as a key step in providing equitable access to high quality care [1-4]. It is also being adopted as an important part of advancing the equity agenda for healthcare systems across jurisdictions [5]. Specifically, in Canada, the Canadian Institutes of Health Research and the Chief Public Health Officer have emphasized the need for the development of tools for health equity measurement [6,7].

In order to identify, track and monitor inequalities across different patient groups, linking patient-level data on health and other outcomes to patient-level sociodemographic data is ideal. Despite increased understanding for the necessity of collecting sociodemographic data for health equity monitoring [3,5] at the system-level, the detailed sociodemographic data that are necessary for monitoring are currently not routinely collected from patients in Canadian healthcare settings and in many other jurisdictions [8-10]. Furthermore, a key data source for linkage to sociodemographic data for health equity monitoring purposes was recently lost, with the cancellation of the mandatory Long-Form Census by the Canadian federal government [11].

A fundamental step to successfully implementing equity measurement is at the level of political will, and in this regard, public opinion represents an important area of investigation. Research in other Canadian jurisdictions (i.e., Calgary, Alberta and British Columbia) has demonstrated general public support for equity-oriented collection of patient-level sociodemographic data [12,13]. However, one of these studies found that there is concern in some Canadian jurisdictions that the collection of ethnicity data will lead to potential harm for racialized or vulnerable groups in the context of healthcare settings [13]. Regional variation in population demographics and perceptions regarding this topic underlines the need to examine public opinion in specific contexts and on specific types of sociodemographic data. In particular, we recently demonstrated significant under-awareness about income-related health inequalities in a sample of Ontarians [14], which may imply less support for the collection of sociodemographic information from patients for purposes of monitoring healthcare inequalities.

Building on a previous study exploring public opinion on this issue with a representative sample of Canadians [5], we undertook a mixed methods study to generate a more in-depth understanding of public opinion on the collection of patient sociodemographic information in the province of Ontario. We first surveyed a representative sample of 1,306 Ontario adults regarding their opinions on the collection of personal information (such as family income, education background, ethnic background and sexual orientation) by hospitals for equity measurement purposes. Since a key limitation of surveys is the lack of opportunity to delve into respondents’ answers to more fully understands their experiences and perceptions, we then conducted a series of in-depth qualitative interviews with a sample of 34 residents of Toronto, Ontario to further explore opinions and validate the survey findings.

## Methods

### Data collection

#### Public opinion survey

Data were collected from a stratified sample of 1,306 Ontarians who were aged 18 years and over at the time of the survey. A sample size calculation indicated that this number of adults could provide a 2.7% margin of error with 95% confidence relative to the population of Ontario. The survey was conducted by telephone interview in November, 2009. A public opinion and market research firm (Harris-Decima) was employed to administer the survey as part of their weekly national omnibus survey. Participants were selected using random digit dialing. In the weeks when our survey was conducted, 72,216 calls were attempted. After excluding numbers that were not in service, fax machines, or invalid, there were 55,205 total eligible calls. For calls with no answer, the system is programmed to automatically call back at another time the next day, or for busy signals, later the same day. After excluding, answering machines, calls with no answer, language barriers, ill or incapable respondents, and no eligible respondent being available, a total of 15,976 people were asked to participate in the survey. Of these, 1,622 people were cooperative contacts, with 1,306 qualifying as eligible and completing the interview. This represents a response rate of 2.9%, with 8.2% of persons asked to complete the survey doing so. Willingness to participate in the survey was taken to imply consent, and no personal identifiers were collected. Surveys were conducted in English and French.

The survey was introduced with a statement suggesting that hospitals may collect personal information from patients “to monitor the quality of the services they provide". Three broad themes were then examined using 5-point Likert scales: [1] agreement with the importance of collecting this type of information (one question), [2] comfort with the collection of information about ethnic background, preferred language, citizenship/immigration status, current household income, educational background and sexual orientation (six questions), and [3] concern that the collection of information could negatively affect care (one question). Participants were also asked to indicate their comfort with five scenarios for the collection of such information: [1] face-to-face reporting with a hospital clerk, [2] face-to-face reporting with a family physician, [3] filling out a form at a hospital, [4] taking a survey by mail or on the internet, and [5] having information accessed from existing government records. Participants were allowed to select more than one option or none at all.

#### In-depth qualitative interviews

In order to further explore opinions and perceptions towards the collection of sociodemographic information in healthcare settings for equity measurement, a sample of 34 individuals who had used healthcare services within the last 12 months and lived in Toronto, Ontario were recruited. Qualitative interview participants did not participate in the Ontario public opinion survey. Recruitment was conducted with the assistance of a public opinion survey research company (Opinion Search). Through their network of databases, the public opinion survey research company recruited, through purposive sampling, healthcare patients from two sociodemographic groups 1) those in mid/high income groups and 2) members of groups who may experience difficulty in accessing quality care and may be most concerned about the collection and (mis)use of personal information in healthcare settings (i.e., low income groups, immigrants and newcomers, members of the LGBTQ community). These groups were sampled in order to capture a broad range of perceptions of the issue [13]. Potential participants were screened for eligibility by the public opinion survey research company and an interview was scheduled to take place at their offices in Toronto. A research assistant from the study team then conducted the interviews. All interviews were conducted in January 2011 and interviews ranged from 30 minutes to 2 hours. Participants were compensated for their participation with a $75 honorarium. All qualitative interviews were audio-recorded with the permission of the study participants.

Participants were asked a series of questions that explored their personal opinions about the collection of sociodemographic data in healthcare settings for the purposes of health equity measurement, their perceptions on the best way to collect these data (should data collection be implemented), as well as their impressions of the findings of the Ontario public opinion survey. Both the survey and interview components of the study were approved by the University of Toronto Research Ethics Board.

### Data analysis

#### Public opinion survey

Descriptive analyses were conducted concerning participants’ place of residence, age, sex, immigrant status, ethnic background, household income, highest attained education level, current employment status, self-rated health, and previous experience with the Canadian health care system. Participants who either had a high school diploma as their highest attained education level, or a household income of under $40,000, or were unemployed at the time of the survey were classified as having low socioeconomic position.

We followed a multi-stage process to determine whether public opinion (i.e., percent disagreed, uncomfortable, or concerned) differed between particular study participant subgroups and to co-adjust multiple subgroup effects. First, we systematically tabulated the proportion of participants that disagreed regarding the importance of data collection, were uncomfortable with the collection of specific data, or were concerned that data collection could negatively impact care across all participant subgroups and identified significant differences based on a chi-square test with an alpha level of 0.05. Second, we examined the Spearman correlation coefficient between all significant subgroup characteristics in order to identify potentially non-independent predictors of public opinion for each outcome to inform the process of model building. Third, we block adjusted for all significant predictors of public opinion for each outcome (as indicated by the chi-square test) in a binary logistic regression model, while avoiding the inclusion of highly colinear predictors. For all survey analyses, data were weighted to replicate provincial population distributions, by age and sex, according to 2006 Census data. SPSS for Windows software (SPSS Inc., version 16.0) was used for all survey analyses.

#### In-depth interviews

Qualitative data were analyzed through a process of data coding involving the constant comparative technique derived from grounded theory methods [15]. Data were coded into common categories based on similar content, and in the process of coding; emerging categories were compared with previous categories. Eventually, a number of broader categories were reduced to a set of higher themes concerning opinions and preferences for the collection of personal information. Data were coded manually by the same research assistant who conducted the interviews, in consultation with the principal investigator of the study.

## Results

### Sample characteristics

The majority of survey participants resided in an urban setting (75%) and 31% were classified as having low socioeconomic position (Table 1). Half of participants were female, while 85% were over the age of 35. The majority of participants were born in Canada (78%), while 4% were recent immigrants (i.e. immigrated less than or equal to 10 years ago). Fifteen percent of survey participants identified as an ethnic or cultural minority.

**Table 1:** Public opinion survey and in-depth interview sample characteristics

	**Survey participants (N = 1,306) -% (#)**	**In-depth interview participants (N = 34) -% (#)**
Residence in a census Metropolitan area^2^		
Yes	75 (974)	34 (100%)
No	25 (332)	0 (0)
Gender		
Male	50 (650)	44 (15)
Female	50 (656)	56 (19)
Age group		
18 to 34	15 (200)	15 (5)
35 to 54	44 (570)	53 (18)
55+	41 (536)	32 (11)
Born in Canada		
Yes	78 (1009)	68 (23)
No (Entry > 10 years ago)	18 (231)	20 (7)
No (Entry < 10 years ago)	4 (49)	12 (4)
Ethnic or cultural minority^3^		
Yes	15 (186)	26 (9)
No	85 (1026)	74 (25)
Low socioeconomic position^4^		
Yes	31 (394)	24 (8)
No	69 (895)	76 (26)

^1^ Columns do not always total to 1306 due to missing values.

^2^ An urban core with a population of at least 100,000 based on the 2006 Canadian Census.

^3^ Did not report Canadian, American or European (including Russian) ethnic ancestry.

^4^ Participant either had high school as their highest attained education level, or a household income of under $40,000, or was unemployed at the time of study participation.

All in-depth interview participants lived in an urban area. Over half of interview participants were female (56%) and the majority were over the age of 35 (85%). Twenty-four percent were classified as having low socioeconomic position. Sixty-eight percent were born in Canada, 12% were recent immigrants, and 26% identified as an ethnic or cultural minority.

### Public opinion survey

#### Importance of sociodemographic data collection for equity measurement purposes

Mixed support for the collection of sociodemographic information in healthcare settings emerged among Ontarians. Overall, 49% of survey participants agreed that it was important for hospitals to collect such information, while 40% disagreed and 11% neither agreed nor disagreed. Variation in support for the collection of patient sociodemographic information was observed across sociodemographic subgroups. Younger participants (18-34 years of age) were less likely to disagree than those 55 years of age and older (OR 0.74, 95% CI 0.55-0.99) Participants of low socioeconomic position were more likely to disagree (OR 1.33, 95% CI 1.04-1.71) (Table 2).

**Table 2:** Percentage of participants disagreeing with, being uncomfortable with or concerned with data collection, and relative disagreement/discomfort/concern in subgroups

	**Disagreement about the importance of collection**	**Discomfort with the specific collection of:**						**Concern that collection could negatively affect care received**
**Study participant subgroup**		**Ethnic background**	**Preferred language**	**Citizenship or immigration status**	**Current household income**	**Education background**	**Sexual orientation**	
	39.9	27.5	6.6	24.4	67.2	38.2	39.6	63.4
	**Odds ratio (95% confidence interval) of relative disagreement/discomfort/concern**^**1**^							
Residence in a census metropolitan area^2^	0.80 (0.61–1.05)							
Ethnic or culturally minority^3^			2.47 (1.42–4.30)^5^					
Age group								
18 to 34	0.74 (0.55–0.99)^5^	0.71 (0.51–0.99)^5^	0.34 (0.17–0.69)^5^					1.47 (1.09–1.98)^5^
35 to 54	1.10 (0.84–1.44)	1.08 (0.81–1.43)	0.66 (0.38–1.13)					1.23 (0.94–1.61)
55+	Reference	Reference	Reference					Reference
Female sex			0.55 (0.33–0.90)^5^			1.23 (0.98–1.55)	1.28 (1.02–1.61)^5^	1.58 (1.25–1.99)^5^
Low socioeconomic position^4^	1.33 (1.04–1.71)^5^							
Birth in Canada (period of immigration)								
Yes						Reference		
No (Entry ≥ 10 years ago)						0.72 (0.53–0.99)^5^		
No (Entry < 10 years ago)						0.48 (0.27–0.87)^5^		

^1^Odds ratio and 95% confidence interval. Odds ratios are reported only for subgroups that were found to be independent, statistically significant predictors of an outcome in the question/column. In these cases, the relative risk is reported after adjusting for other independent predictors of the outcome.

^2^An urban core whose population is at least 100,000 based on the 2006 Census.

^3^Did not report Canadian, American or European (including Russian) ethnic ancestry.

^4^Participant either had a high school diploma as their highest attained education level, or a household income of under $40,000, or was unemployed at the time of survey.

^5^p < 0.05.

#### Level of comfort with disclosing sociodemographic information

While there was a high level of support for the collection of language (only 7% expressed some discomfort), a relatively large proportion of survey participants felt uncomfortable disclosing household income (67%), sexual orientation (40%) and educational background (38%) (Table 2).

There was also variation in comfort with the collection of specific types of sociodemographic information among certain survey participant subgroups (Table 2). Younger participants (18-34 years of age) were less likely to be uncomfortable with the collection of data about ethnic background (OR 0.71, 95% CI 0.51-0.99) and language preference (OR 0.34, 95% CI 0.17-0.69) than those 55 years of age and older. Participants who were ethnic or cultural minorities were more likely to be uncomfortable disclosing their preferred language than non-minorities (OR 2.47, 95% CI 1.42-4.30), while female participants were less likely to be uncomfortable with disclosing language preference than males (OR 0.55, 95% CI 0.33-0.90). In contrast, female participants were more likely to be uncomfortable with disclosing their sexual orientation (OR 1.28, 95% CI 1.02-1.61) than males. Long-term immigrants (i.e., entry into Canada ≥ 10 years ago) were less likely to be uncomfortable with disclosing their education background than those born in Canada (OR 0.72, 95% CI 0.53-0.99), and recent immigrants (i.e., entry into Canada < 10 years ago) were even less likely to be uncomfortable (OR 0.48, 95% CI 0.27-0.87).

The majority of survey participants (63%) were concerned about the potential for misuse of such data (Table 2). Younger participants (18-34 years of age) were more likely to be concerned than those 55 years of age and older (OR 1.47, 95% CI 1.09-1.98), as were female participants compared to males (OR 1.58, 95% CI 1.25-1.99).

#### Suggestions for acceptable information collection methods

As part of the public opinion survey (and in-depth interview), participants were asked, if sociodemographic information were to be collected in healthcare settings, how they would prefer to disclose this information. They were asked if they would prefer to share this information face to face with a hospital clerk, face to face with a physician, filling out a form in a hospital, taking a survey by mail or on the internet, or information could be received through automatic access to existing government records such as health records, driver’s license records and tax records.

**Figure 1:**
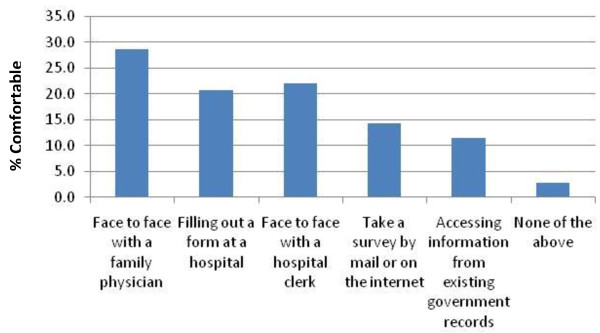
Percent of survey participants comfortable with various approaches for the collection of sociodemographic information from patients.

Twenty-nine percent of survey participants indicated that their preferred method to disclose sociodemographic information would be face to face with a family physician, followed by 22% indicating comfort with disclosing face to face with a hospital clerk, and 20% indicating comfort with disclosing on a form in a hospital (Figure 1). Fourteen percent indicated comfort with disclosing through by survey taken over mail or internet survey, 12% were comfortable with disclosure through existing government records, and three percent indicated comfort with none of these options.

### In-depth interviews

#### Level of comfort with disclosing sociodemographic information

Similar to survey participants, participants in the qualitative interviews expressed feeling most comfortable disclosing language, and least comfortable with disclosing household income, followed by sexual orientation and education. When asked why they and survey respondents might have felt more comfortable with disclosing language in the context of a healthcare visit, interview participants expressed that this was likely the case because it would be important for physicians to know the patient’s language in order to communicate with the patient and deliver optimal care in the context of a personal healthcare visit:

Because… if English isn’t really your first language and you want to speak to a doctor it’s very important that they know that so they can get somebody to…translate or something like that, so it can avoid confusion and frustration…

Interview participants discussed that they were less comfortable with disclosing income and education because they did not think that socioeconomic position should affect immediate healthcare delivery, and expressed concern that such information may be used to judge, pity or even discriminate against the patient.

Interview participants viewed recent immigrants as perhaps more likely to be comfortable with disclosing information about socioeconomic position (e.g., income or education) because they may come from cultures that are more trusting of the need for this information, and/or because of their recent experience with disclosing various types of information during the immigration process, compared to native-born Canadians who were viewed as more protective of their personal information. Interview participants felt that younger individuals would be more comfortable with disclosing various types of sociodemographic information than older individuals due to a generational effect in which youth culture is more open to communication compared to older generations, and tends to be more involved in social networking and information sharing on the internet. Interview participants suspected that females were generally more private and hence more suspicious of how data would be used.

#### Factors influencing disclosure of sociodemographic information

Interview participants were asked to elaborate further on why they and others may or may not be comfortable disclosing sociodemographic information in healthcare settings for equity measurement purposes, and a number of inter-connected themes emerged.

#### Concern for discrimination/stigma

Many participants discussed a concern that disclosure of sociodemographic information, in particular, income, education and sexual orientation, in the context of a healthcare visit may lead to discrimination or judgment on the part of the healthcare provider, and that this would negatively affect their care.

And why do they need to know it?…Now I have 6 or 10 different people knowing about my life… about how much I make, where I went to school because a lot of people, we are in a society where you are judged about who you are, what you do, where you came from, what language do you speak …what do you do for a living and how much do you make.

A number of interview participants felt that disclosure of sexual orientation would lead to discrimination in the context of a healthcare visit, and particularly among members of the lesbian, gay, bisexual, transgendered and queer community. One participant described that a friend felt she had experienced such discrimination while visiting her same sex partner in the hospital.

My good friend actually was just in the hospital a couple of weeks ago and there was myself and her wife there and…she obviously wanted her wife to be there and they actually like refused…apparently that wasn’t okay or something like that to have two women together.

#### Concern regarding relevance of data collection

Interview participants were more open to disclosing such data if there was a sufficient explanation for the use of the information. As mentioned earlier, many assumed that this information would be collected in regard to their immediate care and therefore felt that it would only be necessary if relevant to diagnosis/treatment.

I don’t think it’s important…we’re all people, we all need care…but… maybe that doctor does know more about you know, if there’s certain diseases in, that ethnic background than others… Maybe he is better qualified.

A number of participants questioned why they would need to share such data with healthcare staff.

It depends on why they want it… that would be my first question. But I have probably given all that, information in the hospital, before surgery…automatically because you get these forms thrown at you… and I think I have probably filled them out, just done it… not even thinking, and then I’ve thought afterwards, what the heck did they need my income for? Because they don’t… as far as I am concerned. But give me a good reason you know, tell me why.

One participant articulated that collection of such information would only be justified if background information was needed for immediate care during a healthcare visit or for the purposes of research on health-related issues or conditions.

…Only where it might specifically pertain to the reason the person is seeking medical treatment. …So…on two very narrow contexts do I actually think that information should be gathered. Unless there is a study like this you know, or a study pertaining to something health-related where … on a voluntary basis someone would say you know,… ‘We’re doing a study on… heart attacks you know, are you interested in participating’?

Only a few participants seemed to appreciate the relevance of sociodemographic data collection for monitoring purposes at the population-level, but for many of those participants, they did not see it as relevant specifically for equity measurement purposes, but to understand which populations are accessing the healthcare system.

I think…it gives…a good sense of knowledge of who’s being dealt with. It also gives more statistics I guess in general to know you know, if there’s lower socioeconomic people they’re dealing with, if it’s you know, higher income status and that as well sexual orientation… it might get better statistics overall in terms of who they’re actually tracking…

A number of participants emphasized that it should be made clear to patients that the data will be used for research purposes, and the benefit to Canadians of disclosure should also be clearly explained. Otherwise, some were concerned that their information would be used for reasons of which they would be unaware.

I don’t think I would have a concern about it being misused if I was told up front that this information is being gathered in order to optimize health services, but I certainly would have a concern that this information would be used for purposes other than the venue for which you’re gathering it…

#### Concern for privacy of information

Several interview participants were concerned regarding confidentiality of the personal information that would be collected. For many, this would be a deciding factor on whether to disclose. Over half of the participants were concerned with security measures for the data collected specifically in order to prevent identity theft, and many expressed general concern regarding security and privacy of personal health information in general.

I don’t think that you can guarantee 100% absolutely privacy, I don’t think that’s ever going to happen because there’s breach of privacy all the time…our information is stored on computers, people have the ability to access these computers whether you’ve got 7 passwords… or not, there are always going to be people who can access the information.

I think sometimes too, because… the state of our media… anytime there is any kind of problem or a leak of something, or… they find some medical records in a garbage pail somewhere or something… it’s the front page and…it’s a big story…confidentiality is a huge thing and it needs to be really… regulated in making sure that it’s okay…

#### Suggestions for acceptable information collection methods

The majority of interview participants indicated that their preferred method to disclose sociodemographic information would be face to face with a family physician, should this information be collected. Many interview participants indicated that they would be more comfortable with disclosing this information in the context of an ongoing relationship with a family physician because they could trust such a provider to keep the information private.

I would say the family physician one…I would believe there’s more of a bond between the patient and the doctor whereas it’s not, all these other ones especially the clerk at a hospital…it seems, it’s usually very open when I have been to the hospital, there’s not much privacy so I am telling somebody about me…I want privacy.

#### Need for careful educational messaging to increase comfort

When asked what might lessen the concern over collection and disclosure of this information in healthcare settings, a number of interview participants suggested that an educational campaign would be helpful. This campaign should be very clear in its explanation of the purpose of the information collection and use.

Many participants cautioned that simply saying the information is needed and should be collected is not enough. Some of these participants emphasized that people would want to see how this information is being used, see how it benefits them personally and the population as a whole, and be assured that the information will be used, and not collected, stored and then forgotten. Furthermore, many indicated that the educational campaign should explicitly address security and privacy measures related to use and storage of the information collected. A few participants were unsure as to who should be the voice delivering the campaign message - government or healthcare providers. Some felt that the message should be tailored to specific populations:

…The way that message got framed would have to be really, really well done because… it’s not just enough to say ‘hey everybody it’s important that you share your personal information so that we all get great healthcare’….I think we live in a really skeptical age and…it might have to be not just one general message but it would have to be targeted to different audiences.

Practically speaking, a number of participants indicated that comfort could also be increased if it was assured the data would be collected confidentially and in a format that would prevent the need to repeatedly ask personal characteristic questions at all healthcare visits.

## Discussion

In this study of Ontarians, we found mixed support regarding the importance of individual-level sociodemographic data collection in healthcare settings. Comfort with such data collection appeared to vary across certain participant subgroups. The majority of participants had concerns that the collection of these data could negatively affect their or others’ care. Participants in general were more comfortable providing sociodemographic information to their family physician.

There was substantial variation across participant subgroups in their comfort with the collection of various types of information, but greater discomfort in general for current household income, sexual orientation, and education background. These findings are consistent with other research that has found high rates of non-response for questions such as income and education in health surveys [16], indicating that these are considered to be sensitive questions for the public. However, a recent study also noted significantly lower refusal rates for sexual orientation questions than for household income in American public health surveys [17].

It was briefly explained to interview participants at the beginning and at the end of the in-depth interviews that the purpose of collecting sociodemographic information in healthcare settings was in order to monitor whether patients are receiving equitable and quality care when accessing the healthcare system. Despite these explanations (albeit brief), most participants interpreted the purpose of such data collection to be to assist physicians to deliver proper care in the context of an immediate healthcare visit, as opposed to monitoring equitable access to care at the population-level. While patient sociodemographic information may be used for clinical purposes, this arguably reflects a lack of understanding of a broader purpose of collecting sociodemographic information in healthcare settings to monitor system-level health inequalities. Such a lack of understanding could be related to limited awareness of the social determinants of health and the existence of inequalities in access to quality healthcare among the public, which has been noted in other studies [14,18]. A recent study found that one third of a sample of Ontarians was unaware of income-related inequalities in health, and that there was also lack of awareness of income-related health inequalities in certain health conditions, such as accidents, heart disease and diabetes [14].

Findings from both the public opinion survey and in-depth interviews highlight that there is considerable concern regarding disclosure of personal health information due to anticipated discrimination or negative implications for immediate access to healthcare. These findings have been corroborated in a national study [5] and other studies in particular Canadian jurisdictions [12,13]. However, this study has extended these findings on opinions of health equity measurement techniques further through an in-depth exploration of the nature of this discomfort. There appears to be significant concern among the public regarding the privacy of personal health information, and personal information in general, as well as a strong desire to be made aware of information usage. These results are consistent with other research on the general collection of personal health information. Some of these studies have found that while Canadians are supportive of the use of their personal health information for research that improves public health and quality of care, this support is dependent on the intended use of the data, the data users, and the privacy standards that are applied [19,20]. These concerns are perhaps reflective of concern about privacy standards for the protection of health information, and possibly relate to limited public trust in government use of personal information [21,22]. For example, a public opinion poll in 2007 found that 53% of Canadians felt that they have less protection over their personal information than five years ago [23]. Findings on public opinion on possible strategies for the collection of personal information for health equity monitoring are also consistent with other research in the United States that has found support for the collection of this information by healthcare providers [24]. However, this study, and the study conducted by the authors with a national sample [5], specifically extends existing research in this area in the Canadian context.

Findings from this study thus reveal an interesting paradox related to assumptions about equitable access to healthcare in Canada. The perceived lack of importance of sociodemographic data collection in healthcare settings attributed may be indicative that participants believe access to healthcare in Canada is equitable so there is no need for the collection of this information unless for the purposes of informing immediate care. However, many were concerned that such disclosure would negatively affect their care in the context of a healthcare visit.

The considerable concern regarding disclosure of personal information in healthcare settings and the general lack of awareness of the purpose of such information collection among Ontarians may impede future data collection and subsequently hinder health equity monitoring. While routine collection of sociodemographic information in healthcare settings is only one way to monitor health inequalities, the collection of such information in these settings has become even more important in light of the cancellation of the Long-Form Census by the federal government in Canada, and with the implementation of a voluntary version that may affect the quality of the data. With these changes has come growing concern that health inequalities will widen given a lack of opportunity to effectively monitor them [11]. Furthermore, the collection of this information can assist in monitoring various social determinants of health inequalities, in addition to access to quality healthcare. These issues point to the clear need for increased education for the public about the purpose of sociodemographic data collection as a strategy to address this problem, and information on efforts to maintain patient privacy. It is also necessary that data collection strategies be used that lessen public concerns that disclosure will negatively affect care. Such interventions are logical next steps towards increasing comfort and confidence with the collection of this information for health equity measurement purposes among the public. Furthermore, an examination of the feasibility of different strategies for sociodemographic data collection is needed.

A number of limitations should be considered when interpreting these findings. Public opinion survey sampling involved Ontario residents with telephone landlines. Recent Statistics Canada data show that lower income groups are more likely to have cellular phones, thus our sample may have under-represented those with low incomes [25]. Furthermore, while we attempted to recruit a representative sample of participants for the qualitative interviews, low income and minority groups were slightly under-represented in the sample. The healthcare settings discussed in the public opinion survey and qualitative interviews differed. Public opinion survey participants were asked about their opinions on sociodemographic data collection in hospitals; whereas the qualitative interviews took a broader focus by asking participants their thoughts of such data collection in all healthcare settings. Finally, the qualitative interview sample consisted of individuals who did not complete the public opinion survey. While the mixed methods design adds explanatory power to the study, it would have been ideal if we could have followed up those who had completed the survey to elaborate and explain their responses rather than ask others to interpret the survey findings. Furthermore, unlike the survey sample of Ontarians across the province, interview participants were all residents of Toronto, thus opinions and perceptions captured in the interviews are not representative of all Ontarians.

## Conclusions

This study represents a step in the process towards reducing health inequalities by engaging the public in a policy dialogue, gauging their general support for the collection of sociodemographic information in healthcare settings, and exploring which areas and techniques for equity measurement they deem most important. More research and action is needed to continue to advance this dialogue and move towards better strategies for monitoring and reduction of health inequalities.

## Competing interests

The authors declare that they have no competing interests.

## Authors’ contributions

MK, KS, AL and CQ designed the study. MK and KS conducted analyses and drafted the manuscript. SB conducted the qualitative interviews and analyses. AL critically reviewed the manuscript. CQ supervised analyses and critically reviewed the manuscript. All authors read and approved the final manuscript.

## References

[B1] Agency of Healthcare Research and Quality Measuring healthcare quality 2009 [Online] 2009. [Cited: March 12, 2010.] http://www.ahrq.gov/qual/measurix.htm

[B2] Institute of Medicine, Committee on Understanding and Eliminating Racial and Ethnic Disparities in Health Care Unequal treatment: confronting racial and ethnic disparities in health care 2002 Washington, DC: The National Academic Press

[B3] Krieger N Williams Monitoring socioeconomic determinants for health disparities: tools from the public health disparities Geocoding project Healthcare disparities at the crossroads with healthcare reform 2011 New York: Springer 269 312 R.A. (ed.)

[B4] Weinick RM Measuring racial and ethnic health care disparities in Massachusetts Health Aff 2007 26 1293 1302 10.1377/hlthaff.26.5.1293 17848439

[B5] Lofters A Shankardass K Kirst M Quiñonez C Sociodemographic data collection in Canadian healthcare settings: an examination of public opinion Med Care 2010 49 2 193 199 21150797 10.1097/MLR.0b013e3181f81edb

[B6] Canadian Institutes of Health Research Health equity matters - IPPH strategic plan, 2009-2014 2010 [Online] 2010. [Cited: March 12, 2010.] http://www.cihr-irsc.gc.ca/e/40524.html#4_4

[B7] Public Health Agency of Canada The chief public health Officer's report on the state of public health in Canada 2008 Ottawa: Ontario Minister of Health 2008

[B8] Bierman AS Lurie N Collins KS Addressing racial and ethnic barriers to effective health care: the need for better data Health Aff 2002 21 91 102 10.1377/hlthaff.21.3.91 12026007

[B9] Hasnain-Wynia R Baker DW Obtaining data on patient race, ethnicity and primary language in health care organizations: current challenges and proposed solutions Health Serv Res 2006 41 1501 1518 16899021 10.1111/j.1475-6773.2006.00552.x PMC1797091

[B10] Rodney P Copeland E The health status of black Canadians: Do aggregate racial and ethnic variables hide health disparities? J Health Care Poor Underserved 2009 20 817 823 10.1353/hpu.0.0179 19648707

[B11] Collier R Long-form census change worries health researchers Can Med Assoc J 2010 182 12 E563 E564 10.1503/cmaj.109-3322 20660582 PMC2934824

[B12] Quan H The public endorses collection of ethnicity information in hospital: implications for routine data capture in Canadian health systems Healthcare Pol 2006 1 3 55 64 PMC2585343 19305671

[B13] Varcoe C Harms and benefits: collecting ethnicity data in a clinical context Soc Sci Med 2009 68 1659 1666 10.1016/j.socscimed.2009.02.034 19286294

[B14] Shankardass K Lofters A Kirst M Quiñonez C Public awareness of health disparities by income in Ontario, Canada Int J Equity in Health 2012 11 26 1 10 22613058 10.1186/1475-9276-11-26 PMC3423032

[B15] Glaser BG Strauss AL The discovery of grounded theory: strategies for qualitative research 1967 New York: Aldine de Gruyter

[B16] Turrell G Income non-reporting: Implications for health inequalities research J Epidemiol Community Health 2000 54 207 214 10.1136/jech.54.3.207 10746115 PMC1731636

[B17] VanKim NA Adding sexual orientation questions to state wide public health surveys: New Mexico's experience Am J Publ Health 2010 100 12 2392 2396 10.2105/AJPH.2009.186270 PMC2978164 20966370

[B18] Canadian Population Health Initiative Select highlights on public views on the social determinants of health 2005 Ottawa: Canadian Institute for Health Information

[B19] Willison D Patient consent preferences for research uses of information in electronic medical records: interview and survey data Br Med J 2003 326 1 5 10.1136/bmj.326.7379.1 PMC148897 12586673

[B20] Willison D Alternatives to project-specific consent for access to personal information for health research: what is the opinion of the Canadian public? J Am Med Inform Assoc 2007 14 706 712 10.1197/jamia.M2457 17712084 PMC2213476

[B21] Abelson J Gauvin JP Transparency, trust and citizen engagement: what Canadians are saying about accountability? 2004 Ottawa: Canadian Policy Research Networks

[B22] Abelson J Miller FA Giacomini M What does it mean to trust a health system? a qualitative study of Canadian health care values Health Pol 2009 91 63 70 10.1016/j.healthpol.2008.11.006 19117635

[B23] EKOS Research Associates Electronic health information and privacy survey: what Canadians think - 2007 2007 Ottawa, ON: EKOS Research Associates

[B24] Baker DW Attitudes toward health care providers collecting information about patients' race, ethnicity and language Med Care 2007 45 1034 1042 10.1097/MLR.0b013e318127148f 18049343

[B25] Sciadas G The digital divide in Canada 2002 Ottawa: Statistics Canada, Science, Innovation and Electronic Information Division

